# Hypertension and urologic chronic pelvic pain syndrome: An analysis of MAPP-I data

**DOI:** 10.1186/s12894-024-01407-w

**Published:** 2024-01-28

**Authors:** Rosalynn R.Z. Conic, Terrie Vasilopoulos, Karthik Devulapally, Rene Przkora, Andrew Dubin, Kimberly T. Sibille, Aaron D. Mickle

**Affiliations:** 1 Department of Physical Medicine and Rehabilitation, College of Medicine, University of Florida, Gainesville, FL USA; 2 Department of Orthopaedic Surgery and Sports Medicine, University of Florida, Gainesville, FL USA; 3 Department of Anesthesiology, Division of Pain Medicine, University of Florida, Gainesville, FL USA; 4 Department of Physiological Sciences, College of Veterinary Medicine, University of Florida, PO Box 100144, Gainesville, FL 32610 USA; 5 Department of Biomedical Engineering, College of Engineering, University of Florida, Gainesville, FL USA; 6 Department of Neuroscience, College of Medicine, University of Florida, Gainesville, FL USA

**Keywords:** Urologic chronic pelvic pain syndrome, Interstitial cystitis/bladder pain syndrome, Chronic prostatitis, MAPP, Hypertension, Angiotensin

## Abstract

**Background:**

Urologic chronic pelvic pain syndrome (UCPPS), which includes interstitial cystitis/bladder pain syndrome (IC/BPS) and chronic prostatitis (CP/CPPS), is associated with increased voiding frequency, nocturia, and chronic pelvic pain. The cause of these diseases is unknown and likely involves many different mechanisms. Dysregulated renin-angiotensin-aldosterone-system (RAAS) signaling is a potential pathologic mechanism for IC/BPS and CP/CPPS. Many angiotensin receptor downstream signaling factors, including oxidative stress, fibrosis, mast cell recruitment, and increased inflammatory mediators, are present in the bladders of IC/BPS patients and prostates of CP/CPPS patients. Therefore, we aimed to test the hypothesis that UCPPS patients have dysregulated angiotensin signaling, resulting in increased hypertension compared to controls. Secondly, we evaluated symptom severity in patients with and without hypertension and antihypertensive medication use.

**Methods:**

Data from UCPPS patients (*n* = 424), fibromyalgia or irritable bowel syndrome (positive controls, *n* = 200), and healthy controls (*n* = 415) were obtained from the NIDDK Multidisciplinary Approach to the Study of Chronic Pelvic Pain I (MAPP-I). Diagnosis of hypertension, current antihypertensive medications, pain severity, and urinary symptom severity were analyzed using chi-square test and t-test.

**Results:**

The combination of diagnosis and antihypertensive medications use was highest in the UCPPS group (*n* = 74, 18%), followed by positive (*n* = 34, 17%) and healthy controls (*n* = 48, 12%, *p* = 0.04). There were no differences in symptom severity based on hypertension in UCPPS and CP/CPPS; however, IC/BPS had worse ICSI (*p* = 0.031), AUA-SI (*p* = 0.04), and BPI pain severity (0.02). Patients (*n* = 7) with a hypertension diagnosis not on antihypertensive medications reported the greatest severity of pain and urinary symptoms.

**Conclusion:**

This pattern of findings suggests that there may be a relationship between hypertension and UCPPS. Treating hypertension among these patients may result in reduced pain and symptom severity. Further investigation on the relationship between hypertension, antihypertensive medication use, and UCPPS and the role of angiotensin signaling in UCPPS conditions is needed.

**Supplementary Information:**

The online version contains supplementary material available at 10.1186/s12894-024-01407-w.

## Background

Urologic chronic pelvic pain syndrome (UCPPS) is characterized by persistent pelvic pain for at least three of the preceding six months, in the absence of other causes [1]. In addition to pelvic pain, patients may experience gastrointestinal and neuromuscular symptoms, impaired sexual and reproductive function, and hormonal derangements [2, 3]. Risk factors include a history of infection, chemical irritation, trauma or surgery, and pain may be exacerbated by anxiety, irritability, pain catastrophizing, and depression [4, 5]. While it is considered a common condition, symptomatology is similar to other painful conditions such as irritable bowel syndrome, fibromyalgia syndrome, and inflammatory bowel disease, which may also be comorbid [1]. Consequently, it is likely underdiagnosed, resulting in problems with bladder or urinary tract function, physical functioning, and reduced quality of life. UCPPS can be further subdivided into Chronic Prostatitis-Chronic Pelvic Pain Syndrome (CP/CPPS) and Interstitial Cystitis/Bladder Pain Syndrome (IC/BPS).

CP/CPPS has an estimated worldwide prevalence of 2–10% [6, 7]. It is characterized by prostate inflammation and/or infection, lower abdominal pain, pelvic pain, dysuria, urinary urgency, and frequency. IC/BPS has a higher prevalence among women than men, ranging from 0.045 to 6.5% in women and 0.008-4.2% in men [1, 8, 9]. It can present with severe pelvic pain, loss of bladder control, lower abdominal pain, lower back pain, and painful urination. Current treatment options for UCPPS have limited efficacy and are often associated with undesirable side effects.

Hypertension is often treated with medications that inhibit the renin-angiotensin signaling cascade, including angiotensin-converting enzyme inhibitors (ACE-i) or angiotensin receptor blockers (ARB). The renin-angiotensin-aldosterone system (RAAS) regulates blood pressure and volume. RAAS and activation of angiotensin type 1 receptor also contribute to the local generation of reactive oxidative stress, inflammation, and fibrosis in several organ systems, including the liver, lungs, and heart [10]. 

Little is known about the functional significance of local RAAS signaling in the bladder. Literature demonstrates that angiotensin II (Ang II), the signaling peptide in RAAS that activates angiotensin type 1 receptor, is produced locally in human and rat bladder tissue [11–14]. In pre-clinical models, angiotensin receptor type 1 and type 2 are expressed in the bladder, and activation of type 1, can induce detrusor contraction in pre-clinical models [15–17]. 

However, there are several intriguing links between IC/BPS pathology and RAAS, particularly Ang II signaling IC/BPS patients have increased mast cells in their bladder [18–20], representing a potential source of increased renin and Ang II [21, 22]. IC/BPS patients and animal disease models have increased oxidative stress in the bladder [23–30], and angiotensin signaling increases oxidative stress [31]. IC/BPS patients have increased expression of inflammatory mediators, which Ang II downstream signaling can release [1, 32–36]. 

Fibrosis is observed in patients with IC/BPS [18–20, 37–41], and Ang II signaling has been linked to fibrosis in heart [42], lungs [10], liver [43], and kidneys [44].

Therefore, we hypothesized that diseases with modified angiotensin signaling (hypertension) may have greater symptomology compared to normotensive patients and that patients treated with angiotensin modulators would have lower disease scores compared to those not on angiotensin modulators.

Herein we aim to understand the relationship between hypertension and UCPPS, and evaluate if there is an association between angiotensin signaling drugs and UCPPS symptom severity measures using cross-sectional data from the Multidisciplinary Approach to the Study of Chronic Pelvic Pain I (MAPP- I) Research Network [45]. 

## Methods

The Multidisciplinary Approach to the Study of Chronic Pelvic Pain Research Network, which consists of multiple investigators across various fields, including urology, gynecology, rheumatology, gastroenterology, epidemiology, biology, and psychology was established by the National Institute of Health to evaluate the urological, non-urological, and psychosocial symptoms of patients suffering from UCPPS. This study uses data from the Epidemiology and Phenotyping (EP) study from the first phase of MAPP, also known as MAPP I [45]. Patients were enrolled from six sites in the United States over a three-year period (from December 14, 2009 through December 14, 2012, *n* = 1,039) [46]. Data were obtained from MAPP following approval by the University of Florida Institutional Review Board (IRB#202,200,510).

### Participants

The study sample included healthy controls with no concomitant pain conditions (*n* = 415), positive controls with non-urologic (including fibromyalgia, chronic fatigue syndrome, and irritable bowel syndrome, *n* = 200), and patients with UCPPS (*n* = 424).

### Measures

#### Demographics

During a baseline phenotyping visit, information including age, gender, ethnicity/race, educational level, current employment, and income was collected.

#### Health history and medications

Study participants’ health history, list of prescriptions and over-the-counter medications, and physical exam with vital signs, including blood pressure, were also collected during the baseline session.

#### Diagnosis of hypertension

Patients were identified as hypertensive based on self-reported diagnosis or a history of medications traditionally used to treat hypertension (ACE-i/ARB, beta-blockers) (Supplemental Table 1).

#### Symptom measures

Measures that indicate UCPPS symptom severity with consideration for variable reduction were selected.

Interstitial Cystitis Symptom Index (ICSI) and Interstitial Cystitis Problem Index (ICPI) [47]: The ICSI assesses the frequency of symptoms, and the ICPI assesses the impact of symptoms over the previous month, including bladder pain, urgency, frequency, and nocturia. ICSI scores range from 0 to 19 with higher scores indicating greater symptoms. ICPI scores range from 0 to 16 with higher scores indicating greater impact of symptoms. Measures in the analyses include the ICSI bladder pain symptom total (0–4 score) and the ICPI problem severity (0–4 score).

American Urological Association Symptom Index (AUA-SI) [48]: The AUA-SI consists of seven questions assessing urinary symptoms, including urgency, frequency, and voiding over the previous month on a 6-point Likert scale from 0 (“never”) to 5 (“almost always”). Total scores range from 0 to 35 with higher scores indicating more severe urinary symptomatology.

Genitourinary Pain Index (GUPI) [49]: The GUPI assesses genitourinary pain severity (PS), urinary symptoms (US), and quality of life (QOL) over the previous week. Scoring consists of three subscales, pain severity (0–23), urinary symptom severity (0–10), and quality of life impact (0–12) with total scores ranging from 0 to 45. Higher scores indicate greater pain, urologic symptoms, and worse quality of life. The three subscales and total score were included in the analyses.

Brief Pain Inventory (BPI) [50]: The BPI consists of a body map to indicate the areas of pain in the past 24 h. Measures of pain severity (average of worst, least, average, and right now) on a 0-no pain to a 10- pain as bad as you can imagine scale were collected. Pain interference is assessed specifically regarding general activity, mood, walking ability, normal work, relationships, sleep, and enjoyment of life from 0 – does not interfere to 10 – completely interferes. Pain medications and relief from pain medication in the past 24 h are also assessed. Pain severity and pain interference scores are included in the analyses.

### Statistical analysis

Continuous measures were summarized as means and standard deviations and categorical measures were summarized as frequencies and percentages. Hypertension-related measures (hypertension diagnosis, anti-hypertension medication use) were compared across MAPP cohorts with chi-square tests. In the UCCPS cohort only, differences in pain related outcomes were compared between those with and without hypertension diagnosis [49], using t-tests. In the UCCPS cohort, participants with diagnosed hypertension, pain-related outcomes were compared between those who reported taking antihypertensive medication and those that did not, using Mann-Whitney tests. Subgroup analysis of CP/CPPS and IC/BPS was performed. Hypertensive patients who were not on medication were age-matched to patients using antihypertensives. *P* < 0.05 was considered statistically significant. Analyses were conducted in JMP Pro 16 (SAS Institute Inc, Cary NC) and R statistical software (4.2.3).

## Results

There were 415 healthy controls, 200 positive controls, and 424 UCPPS patients (Table 1). The average age was 40.5 ± 14.1 for healthy controls, 41.7 ± 13.7 years for positive controls, and 43.4 ± 15.1 for UCPPS. Positive controls were mostly female (78% vs. 56.1% for healthy controls and 55% for UCPPS). All three cohorts were predominantly white (healthy controls 76.1%, positive controls 75%, and UCPPS 88.2%) of non-Hispanic ethnicity (91.6%, 93%, and 93%, respectively). Educational levels were similar across the three groups; however, unemployment rates were lowest in the UCPPS group (14% vs. 21% for controls). UCPPS patients were further subdivided into CP/CPPS (11.3%, *n* = 48), IC/BPS (55.2%, *n* = 234), and both (33.5%, *n* = 142) (Table 2**)**.

**Table 1:** Demographic characteristics of healthy controls, positive controls and urologic chronic pelvic pain syndrome patients

	Healthy Controls	Positive Controls	UCPPS		*p*-value healthy controls vs. UCPPS	*p*-value
Measure	*n* = 415	*n* = 200	*n* = 424			
Age (mean (SD))	40.51 (14.07)	41.72 (13.74)	43.37 (15.11)		0.004	0.02
Sex					0.73	< 0.001
Female	233 (56.1)	156 (78.0)	233 (55.0)			
Male	182 (43.9)	44 (22.0)	191 (45.0)			
Race (%)					< 0.001	< 0.001
Asian	24 (5.8)	8 (4.0)	9 (2.1)			
Black	48 (11.6)	22 (11.0)	16 (3.8)			
Multi Race	12 (2.9)	10 (5.0)	11 (2.6)			
Native Hawaiian	0 (0.0)	1 (0.5)	0 (0.0)			
Other	11 (2.7)	8 (4.0)	11 (2.6)			
Unknown	4 (1.0)	1 (0.5)	3 (0.7)			
White	316 (76.1)	150 (75.0)	374 (88.2)			
Ethnicity (%)					0.61	0.55
Hispanic	35 (8.4)	13 (6.5)	28 (6.6)			
Not Hispanic	380 (91.6)	186 (93.0)	395 (93.2)			
Unknown	0 (0.0)	1 (0.5)	1 (0.2)			
Education (%)					0.93	0.66
< High School	2 (0.5)	1 (0.5)	0 (0.0)			
High School or GED	27 (6.5)	9 (4.5)	31 (7.3)			
Some college	115 (27.7)	66 (33.0)	118 (27.8)			
College/university graduate	154 (37.1)	72 (36.0)	163 (38.4)			
Graduate or professional school	117 (28.2)	52 (26.0)	112 (26.4)			
Employment (%)					< 0.001	< 0.001
Disabled	0 (0.0)	25 (12.5)	32 (7.5)			
Employed	294 (70.8)	116 (58.0)	278 (65.6)			
Full time homemaker	7 (1.7)	7 (3.5)	12 (2.8)			
Retired	27 (6.5)	8 (4.0)	43 (10.1)			
Unemployed	86 (20.7)	42 (21.0)	58 (13.7)			
Income (%)					< 0.001	< 0.001
$10,000 or less	44 (10.6)	29 (14.5)	42 (9.9)			
$10,001 to $25,000	56 (13.5)	33 (16.5)	34 (8.0)			
$25,001 to $50,000	112 (27.0)	49 (24.5)	69 (16.3)			
$50,001 to $100,000	120 (28.9)	46 (23.0)	122 (28.8)			
Greater than $100,000	53 (12.8)	28 (14.0)	120 (28.3)			
Prefer not to answer	30 (7.2)	15 (7.5)	37 (8.7)			
UCPPS Type						
CP/CPPS			48 (11.3)			
IC/BPS			234 (55.2)			
Both			142 (33.5)			
Self-Reported Hypertension*					0.32	0.21
No	373 (90.3)	171 (85.5)	372 (88.1)			
Yes	40 (9.7)	29 (14.5)	50 (11.9)			
Antihypertensive Use (%)**					0.61	0.87
No	218 (84.5)	146 (83.4)	322 (83.0)			
Yes	40 (15.5)	29 (16.6)	66 (17.0)			
Used ACE-I (%)***	21 (52.5)	14 (48.3)	20 (30.3)		0.02	0.252
Used ARB (%)***	2 (5)	0 (0.0)	15 (22.7)		0.02	0.002
Self-Reported Hypertension and/or Antihypertensive Use (%)***					0.02	0.04
No	367 (88.4)	166 (83)	350 (82.5)			
Yes	48 (11.6)	34 (17.0)	74 (17.5)			

* Not all patients had available data. Healthy controls *n* = 413; Positive Controls *n* = 200; UCPPS *n* = 422

** Not all patients had available data. Healthy controls *n* = 258; Positive Controls *n* = 175; UCPPS *n* = 388

*** Denominator used was Antihypertensive use-Yes

**** This number represents a composite of patients who self-reported diagnosis of hypertension and/or use of antihypertensives

Abbreviations: UCPPS: Urologic chronic pelvic pain syndrome; ACE-i: Angiotensin Converting Enzyme Inhibitors; CP/CPPS: Chronic Prostatitis-Chronic Pelvic Pain Syndrome; IC-PBS: Interstitial Cystitis/Bladder Pain Syndrome

**Table 2:** Demographic characteristics of Chronic Prostatitis-Chronic Pelvic Pain Syndrome and Interstitial Cystitis-Bladder Pain Syndrome patients

	CP/CPPS	IC/BPS	*p*-value
Measure	*n* = 48	*n* = 234	
Age (mean (SD))	45.44 (14.22)	40.51 (14.33)	0.031
Sex			< 0.001
Female	0 (0.0)	233 (99.6)	
Male	48 (100.0)	1 (0.4)	
Race (%)			0.388
White	45 (93.8)	205 (87.6)	
Asian	2 (4.2)	4 (1.7)	
Black	0 (0.0)	7 (3.0)	
Multi Race	0 (0.0)	10 (4.3)	
Native Hawaiian	0 (0.0)	0 (0.0)	
Other	1 (2.1)	6 (2.6)	
Unknown	1 (0.7)	1 (0.5)	
Ethnicity (%)			0.083
Hispanic	3 (6.2)	18 (7.7)	
Not Hispanic	44 (91.7)	216 (92.3)	
Unknown	1 (2.1)	0 (0.0)	
Education (%)			0.018
< High School	0 (0.0)	0 (0.0)	
High School or GED	2 (4.2)	17 (7.3)	
Some college	6 (12.5)	76 (32.5)	
College/university graduate	22 (45.8)	87 (37.2)	
Graduate or professional school	18 (37.5)	54 (23.1)	
Employment (%)			0.027
Employed	40 (83.3)	145 (62.0)	
Full time homemaker	0 (0.0)	12 (5.1)	
Unemployed	4 (8.3)	39 (16.7)	
Disabled	0 (0.0)	24 (10.3)	
Retired	4 (8.3)	13 (5.6)	
Income (%)			0.016
$10,000 or less	3 (6.2)	32 (13.7)	
$10,001 to $25,000	2 (4.2)	22 (9.4)	
$25,001 to $50,000	6 (12.5)	43 (18.4)	
$50,001 to $100,000	11 (22.9)	61 (26.1)	
Greater than $100,000	23 (47.9)	53 (22.6)	
Prefer not to answer	3 (6.2)	23 (9.8)	
Self-Reported Hypertension*			0.96
No	44 (91.7)	214 (91.4)	
Yes	4 (8.3)	20 (8.6)	
Antihypertensive Use (%)**			0.39
No	28 (82.4)	192 (87.7)	
Yes	6 (17.6)	27 (12.3)	
Used ACE-I (%)***	2 (33.3)	9 (33.3)	0.98
Used ARB (%)***	2 (33.3)	5 (18.5)	0.526
Self-Reported Hypertension and/or Antihypertensive Use (%)****			0.89
No	42 (87.5)	203 (86.8)	
Yes	6 (12.5)	31 (13.2)	

* Not all patients had available data

** Not all patients had available data

*** Denominator used was Antihypertesnive use-Yes

**** This number represents a composite of patients who self-reported diagnosis of hypertension and/or use of antihypertensives

Abbreviations: CP/CPPS: Chronic Prostatitis-Chronic Pelvic Pain Syndrome; IC-PBS: Interstitial Cystitis/Bladder Pain Syndrome; ACE-i: Angiotensin Converting Enzyme Inhibitors

### Incidence of hypertension

Positive controls had the highest incidence of hypertension (*n* = 29, 15%), followed by UCPPS (*n* = 50, 12%) and healthy controls (*n* = 40, 10%); however, this was not statistically significant (*X*^2^(2, *n* = 1,035) = 3.16, *p* = 0.21). Within UCPPS, 8.3% (*n* = 4) with CP/CPPS and 8.6% (*n* = 20) with IC/BPS had a diagnosis of hypertension (*X*^2^(1, *n* = 282) = 0.002, *p* = 0.96)

Use of antihypertensive medications was similar across the three groups (*n* = 40, 16% healthy controls vs. 17% in positive controls (*n* = 29) and UCPPS (*n* = 66) (*X*^2^(2, *n* = 821) = 0.26, *p* = 0.87). Among UCPPS patients, 19% (*n* = 6) of CP/CPPS and 14% (*n* = 27) of IC/BPS patients used antihypertensive medication (*X*^2^(1, *n* = 253) = 0.73, *p* = 0.39)

The combination of diagnosis and use of antihypertensive medications was highest in the UCPPS group (*n* = 74, 18%), followed by positive controls (*n* = 34, 17%) and healthy controls (*n* = 48, 12%), (*X*^2^(2, *n* = 1,039) = 6.46, *p* = 0.04). Of CP/CPPS patients, 12.5% (*n* = 6) had a diagnosis or were on antihypertensives, and 13.2% (*n* = 31) of IC/BPS (*X*^2^(1, *n* = 282) = 0.02, *p* = 0.89)

The incidence of angiotensin receptor blockers (ARB) use was greatest among UCPPS group (4%, *n* = 15) vs. 1% (*n* = 2) for healthy controls, 0% for positive controls, *p* = 0.002); while the rates of ACE inhibitor (ACE-i) use was similar (8% for each control group, 5% UCPPS) (*X*^2^(2, *n* = 821) = 2.81, *p* = 0.25)

When comparing healthy controls to UCPPS patients, ARB use (*p* = 0.02), along with combined self-reported hypertension/antihypertensive use (*p* = 0.02) were significantly higher in the UCPPS group, while ACE-I use was higher among healthy controls (*p* = 0.02)

### Hypertension and UCPPS symptom severity

**Fig. 1:**
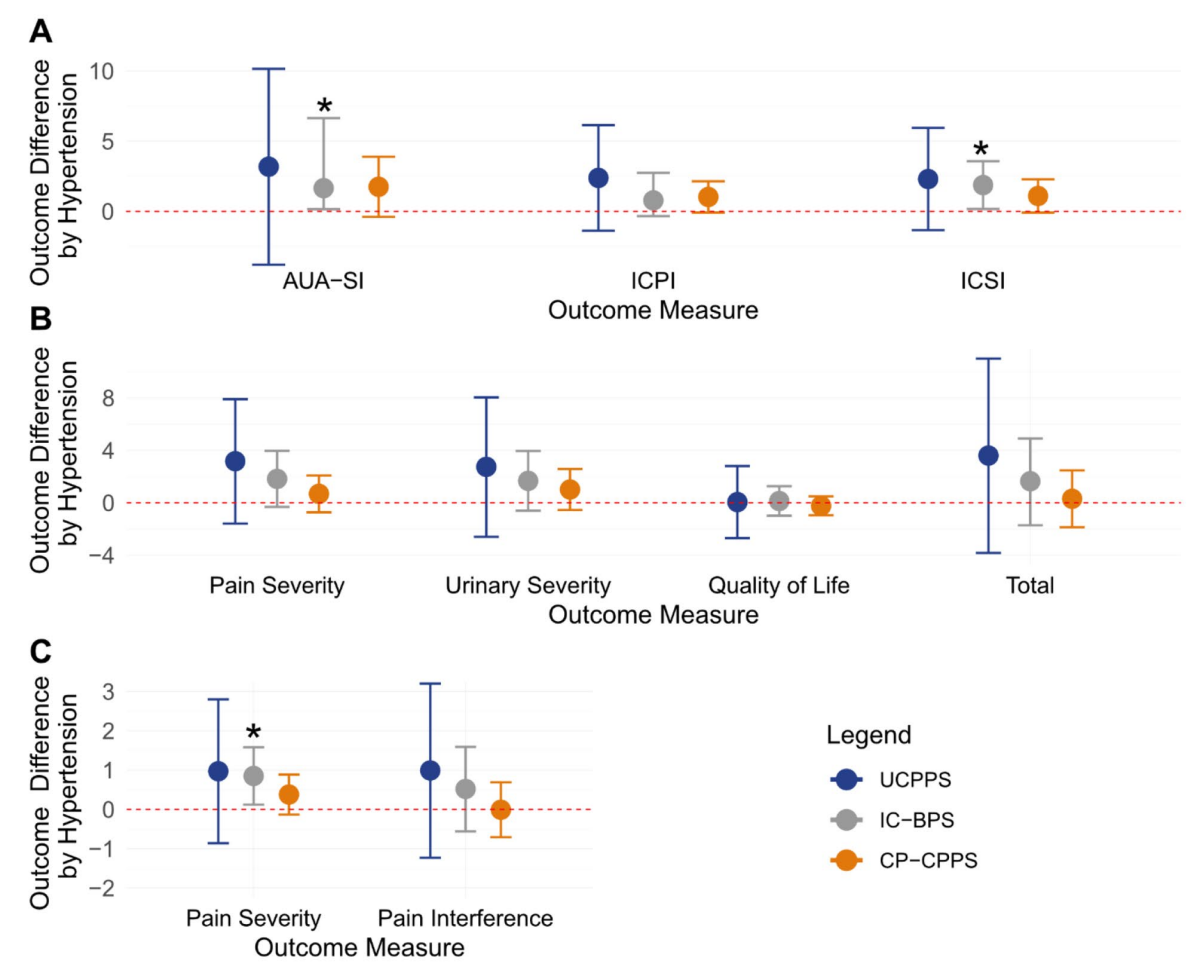
ICSI, AUA-SI, and BPI pain severity were significantly higher in IC/BPS patients with hypertension. Differences in (**A**) ICSI, AUA-SI, and (**C**) BPI based on hypertension. The circle represents the mean and the error bars represent 95% confidence intervals. Black stars denote significant differences between hypertensive and normotensive patients. Confidence intervals that cross the dotted red line are considered non-significant

Diagnosis of hypertension was not significantly associated with symptom severity in UCPPS with consideration of the following measures: ICSI, ICPI, AUA-SI, GUPI (QOL, pain severity, urinary severity, and total score), or BPI (pain severity and interference, Fig. 1A-C). Similarly, there were no differences in pain-related outcomes based on hypertension among CP/CPPS.

However, patients diagnosed with hypertension and IC/BPS had significantly worse symptom severity based on the ICSI (difference 1.87, 95% CI 0.17–3.57, *p* = 0.031), AUA-SI (1.65, 95% CI 0.16–6.64, *p* = 0.04), and BPI Pain severity score (0.85, 95% CI 0.12–1.58, *p* = 0.022), but not ICPI, BPI Pain interference or GUPI (QOL, pain severity, urinary severity, and total score).

### Use of antihypertensive medications and symptom severity

**Fig. 2:**
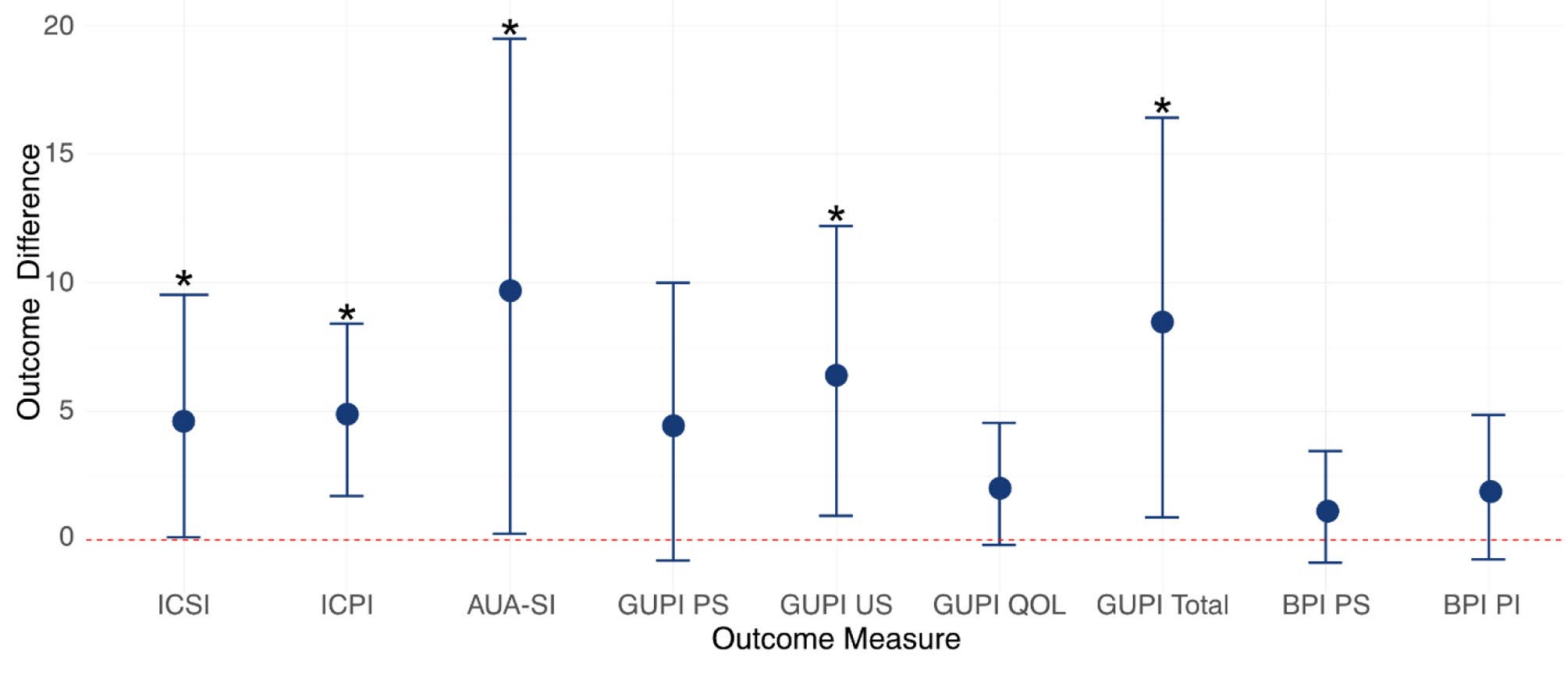
ICSI, ICPI, AUA-SI, GUPI urinary severity, and total score were significantly higher for hypertensive UCPPS patients not on antihypertensives compared to those who were. Differences in ICSI, ICPI, AUA-SI, GUPI, and BPI outcomes among hypertensive UCPPS patients based on antihypertensive medication use. The circle represents the mean, and the error bars represent 95% confidence intervals. Black stars denote significant differences between hypertensive and normotensive patients. Confidence intervals that cross the dotted red line are considered non-significant

Age-matched patients who were diagnosed with hypertension, but were not using antihypertensives (*n* = 7) had higher symptom severity specific to ICSI (*p* = 0.04), ICPI (*p* = 0.02), AUA-SI (*p* = 0.03), GUPI urinary severity (*p* = 0.035), total GUPI (*p* = 0.037), while GUPI QOL impact (*p* = 0.08) trended toward significance (Fig. 2). There were no significant differences in GUPI pain severity (*p* = 0.12), BPI pain severity (*p* = 0.22), or pain interference (*p* = 0.35). There were also no differences in symptom severity based on the use of ACE-i/ARB compared to other antihypertensive medications. Of age matched patients with hypertension who were not using antihypertensives, 4 had IC/BPS, and 3 had CP/CPPS. In addition, of these patients, 5 of 7 had elevated blood pressures (> 130/80) at the time of initial evaluation.

## Discussion

In this study, we evaluated the relationship between hypertension and UCPPS, and whether a diagnosis of hypertension and/or use of antihypertensive medications is related to symptom severity. As the sample sizes were small, findings warrant interpretation as preliminary and do not demonstrate a cause and effect relationship. We observed a non-significant trend suggesting the incidence of hypertension was higher among positive controls and UCPPS patients compared to healthy controls. Additionally, ARB use was greater among the UCPPS group compared to positive and healthy controls. Hypertension was associated with higher symptom severity among patients with IC/BPS, but not UCPPS in general or CP/CPPS alone. Furthermore, the seven UCPPS individuals with hypertension who were not on antihypertensives reported significantly worse symptom severity compared to those receiving treatment. Finally, we did not detect a difference in symptom severity based on the use of ACE-i/ARB.

### Hypertension as a comorbidity of UCPPS

Among the MAPP-I cohort, 18% of UCPPS patients (12.5% CP/CPPS and 13.2% IC/BPS), 17% of positive controls, and 12% of healthy controls were diagnosed with hypertension or used antihypertensive medication; however, there were no significant differences between these groups. While studies evaluating hypertension in UCPPS are minimal, our findings regarding CP/CPPS are similar to those reported in the Health Professionals Follow Up study, where no differences in hypertension were noted after adjustment for smoking and BMI [51]. Similarly, a study of male participants in China found no difference in the prevalence of hypertension in those diagnosed with CP/CPPS compared to controls [52]. In contrast, Pontari et al. demonstrated an 11% lifetime prevalence of cardiovascular diseases among CP/CPPS patients compared to 2% in age-matched controls; however, while they note hypertension was the most common condition, they do not distinguish further [4]. Similarly, a study from Taiwan found that CP/CPPS patients had a higher prevalence of hypertension compared to controls [53]. 

Also completed in Taiwan, a study of patients with IC/BPS found no difference in rates of hypertension between patients with IC/BPS and controls [54]. Similarly, in a study of Brazilian individuals, there were no differences in self-reported hypertension [55]. In contrast, a recent study from China found that the prevalence of hypertension in IC/BPS was 30.8% compared to 13.2% among age and parity-matched controls [56]. Another study similarly found a greater prevalence of hypertension among subjects with IC/BPS compared to controls [57]. The greater prevalence of hypertension in UCPPS patients compared to controls without a significant difference could be due to the smaller sample size represented in this cohort. Furthermore, hypertension prevalence in the US is estimated to be 45.4%, which is significantly higher compared to the controls in our study, potentially due in part to the age representation, female predominance, and greater educational level, all of which are associated with lower rates of hypertension [58]. 

### Use of antihypertensives in UCPPS patients and controls

Next, among the MAPP-I cohort, 4% of UCPPS patients used ARBs, and 5% used ACE-i, which is in contrast to the healthy controls where 1% used ARBs and 8% used ACE-i. This finding among UCPPS patients also differs from the literature where ACE-i are more commonly prescribed [59]. 

### Hypertension and UCPPS symptom severity

Hypertension was associated with worse symptom severity in IC/BPS patients but not CP/CPPS or overall UCPPS patients. No differences were noted in symptom severity based on the medication used (Fig. 1). Similarly, a study stratifying CP/CPPS by intensity of pain did not find hypertension to be a significant risk factor [60]. It is important to note that in the general public, only 40% of patients on antihypertensives have well-controlled blood pressure. It is also possible that despite taking an antihypertensive, the medication is insufficient to control deregulated hormones and does not contribute to symptom severity outcomes [61]. 

### Antihypertensives and UCPPS symptom severity

Finally, although interpretation is limited by the sample size, age-matched UCPPS hypertensive patients not on anti-hypertensives had worse symptom severity compared to those who were treated with antihypertensives, which has not been reported previously. Prior studies evaluating the relationship between pain, which is the most common domain within the symptom severity outcomes, and hypertension had mixed results. Some studies demonstrate a positive relationship between pain and hypertension, some demonstrate decreased pain perception, while others demonstrate no association [62]. It is possible that untreated, hypertensive patients have greater levels of angiotensin II and aldosterone which can further potentiate inflammation, dysregulate the hypothalamic-pituitary-adrenal axis, and contribute toward worse symptom severity [63]. 

For example, Dimitrakov et al. evaluated 27 CP/CPPS patients and 20 age matched asymptomatic controls and found that CP/CPPS patients, in general had lower corticosterone and aldosterone levels, and higher progesterone levels compared to controls [64]. However, in CP/CPPS patients, higher NIH-CPSI total and pain domain scores (indicating worse symptom severity) were associated with greater aldosterone levels [64]. Prior mouse studies demonstrated that aldosterone production leads to hypersensitivity and likely contributes to pain signaling [65]. 

In regards to ACE-i compared to ARBs, our UCPPS cohort tended to use ACE-i more than healthy controls. While this could be a coincidence, ACE-i may be implicated in decreased breakdown of bradykinin and it is possible that these patients exhibited more side-effects from ACE-i use [66]. However, the sample size was not large enough to further differentiate the effects between ACE-i, ARB, and other antihypertensives.

### Limitations and future directions

There are several limitations to acknowledge. First, this study is limited by a small sample size and can only be considered a preliminary investigation. By definition of a retrospective cohort, the study design is unable to demonstrate a cause and effect relationship, only associations. There is also limited minority ethnic and race group representation in this sample, further limiting generalizability to the diverse U.S. population.

Next, self-reported diagnosis of hypertension is subject to recall bias, and these patients could be misclassified. In addition, while we eliminated medications not traditionally used as antihypertensives, it is possible that individuals are misclassified as having hypertension based on their medication use. Conversely, it is also possible that some patients are using non-traditional medications for their hypertension and are misclassified as normotensive. Furthermore, it is possible patients developed hypertension at a later time period from when the initial evaluation was completed. In addition, we do not have longitudinal medication data to determine whether individuals were started on additional pain medications, on another antihypertensive medication, and/or if medications were stopped or switched.

Third, the reported rates of hypertension are significantly lower compared to the general population across all three cohorts, possibly indicating a lack of external generalizability. It is also unknown why the prevalence of ARB use was significantly higher in the UCPPS group compared to the other two groups, although it may be related to hypertension control. Fourth, of the patients who are considered hypertensive, who were not on medication, two of seven did not have elevated blood pressure (systolic ≥ 130mmHg and/or diastolic ≥ 80mmHg) at the baseline visit, meaning that their hypertension may be controlled by lifestyle modifications. Finally, hypertension is only one factor which may contribute to the patient’s experience of pain- elevated blood glucose, presence of inflammation, obesity and many others were not included and should be evaluated in future studies.

While this study is among the largest evaluating the incidence of hypertension in UCPPS, and evaluating the impact of hypertension on symptom severity, sample sizes to address the questions of interest were small. We were limited in identifying the relationship between symptom severity and specific medications, and whether hypertension control helps regulate pain severity. Furthermore, prospectively evaluating whether there is improvement in pelvic pain symptoms for hypertensive UCPPS patients when they are placed on ACE-i or ARB compared to other pain management strategies would be an important next step. Further investigation in these areas is needed. If these relationships prove true, treating UCPPS patients with antihypertensive medications as part of the treatment regimen might help reduce pain and improve symptom management.

## Conclusion

UCCPS may be co-morbid to hypertension or the use of antihypertensive drugs. The use of antihypertensive medications was associated with a reduction in symptoms and pain severity in a small sample. Based on pre-clinical data and our preliminary findings, further investigations with larger sample sizes on the relationship between hypertension and UCPPS and the effects of different medications on symptom severity are needed.

### Electronic supplementary material

Below is the link to the electronic supplementary material.

Supplementary Material 1

## Data Availability

Data from the Multidisciplinary Approach to the Study of Pelvic Pain [(V4)/10.58020/emxg-8065] reported here are available for request at the NIDDK Central Repository (NIDDK-CR) website, Resources for Research (R4R), https://repository.niddk.nih.gov/.

## Abbreviations

UCPPS: Urologic chronic pelvic pain syndrome
IC/BPS: Interstitial cystitis/bladder pain syndrome
CP/CPPS: Chronic prostatitis
ACE-i: Angiotensin-converting enzyme inhibitors
ARB: Angiotensin receptor blockers
ICSI: Interstitial Cystitis Symptom Index
ICPI: Interstitial Cystitis Problem Index
AUA-SI: American Urological Association Symptom Index
GUPI: Genitourinary Pain Index
QOL: Quality of life
BPI: Brief Pain Inventory
MAPP: Multidisciplinary Approach to the Study of Chronic Pelvic Pain I
RAAS: Renin-Angiotensin-Aldosterone-System
Ang II: Angiotensin II
