# Supplementary material for: Hypertension and urologic chronic pelvic pain syndrome: An analysis of MAPP-I data
Source: BMC Urol. 2024 Jan 28;24:21. doi: 10.1186/s12894-024-01407-w (PMC10822153; doi:10.1186/s12894-024-01407-w)
Supplement: Supplementary file 1 — Supplementary Material 1 [file 12894_2024_1407_MOESM1_ESM.docx]

**SUPPLEMENTAL INFORMATION**

Supplemental Table 1. List of Antihypertensive Medications

| NAME | Type of Medication |
| --- | --- |
| Aliskiren Hemifumarate | ACE-i |
| Amlodipine Besylate/Benazepril | ACE-i |
| Benazepril Hcl | ACE-i |
| Enalapril Maleate | ACE-i |
| Enalapril/Hydrochlorothiazide | ACE-i |
| Fosinopril Sodium | ACE-i |
| Lisinopril | ACE-i |
| Lisinopril/Hydrochlorothiazide | ACE-i |
| Ramipril | ACE-i |
| Trandolapril | ACE-i |
| Amlodipine/Valsartan | ARB |
| Irbesartan | ARB |
| Losartan Potassium | ARB |
| Losartan/Hydrochlorothiazide | ARB |
| Olmesartan Medoxomil | ARB |
| Olmesartan/Hydrochlorothiazide | ARB |
| Telmisartan | ARB |
| Valsartan | ARB |
| Valsartan/Hydrochlorothiazide | ARB |
| Amlodipine Besylate | Other |
| Atenolol | Other |
| Carvedilol | Other |
| Chlorthalidone | Other |
| Clonidine | Other |
| Diltiazem Hcl | Other |
| Doxazosin Mesylate | Other |
| Eplerenone | Other |
| Furosemide | Other |
| Guanfacine Hcl | Other |
| Hydrochlorothiazide | Other |
| Labetalol Hcl | Other |
| Metipranolol | Other |
| Metoprolol Succinate | Other |
| Metoprolol Tartrate | Other |
| Metoprolol/Hydrochlorothiazide | Other |
| Nadolol | Other |
| Nebivolol Hcl | Other |
| Prazosin Hcl | Other |
| Propranolol Hcl | Other |
| Spironolactone | Other |
| Timolol Maleate | Other |
| Torsemide | Other |
| Triamterene | Other |
| Triamterene/Hydrochlorothiazide | Other |
| Verapamil Hcl | Other |
